# Enhancing Medical Diagnosis Document Analysis with Layout-Aware Multitask Models

**DOI:** 10.3390/diagnostics15233039

**Published:** 2025-11-28

**Authors:** Hung-Jen Tu, Jia-Lien Hsu

**Affiliations:** Department of Computer Science and Information Engineering, Fu Jen Catholic University, New Taipei City 24205, Taiwan; danghoangnhan.1@gmail.com

**Keywords:** Optical Character Recognition, Key Information Extraction, document understanding

## Abstract

**Background and Objectives:** Medical diagnosis documents often exhibit diverse layouts and formats, posing significant challenges for automated information extraction. Ensuring the privacy of sensitive medical data further complicates the development of effective analysis systems. This study aims to develop a robust and privacy-compliant system for analyzing medical diagnosis documents. **Methods:** We designed an integrated Optical Character Recognition (OCR) system that processes medical documents regardless of their layout or format. The system first converts bitmap images into machine-readable text using OCR. A document-understanding model is then applied to identify and extract key information. To improve adaptability and accuracy, we employed a mutual learning approach. To address privacy concerns, we generated training data using generative techniques, ensuring compliance with privacy regulations while maintaining dataset quality. **Results:** The proposed system demonstrated strong performance across a wide variety of document layouts, effectively extracting critical information while adhering to privacy requirements. **Conclusions:** Our approach offers a practical and efficient solution for processing complex medical diagnosis documents, advancing the field of medical informatics while safeguarding patient privacy.

## 1. Introduction

The healthcare industry has risen to a significant challenge in managing the vast amount of unstructured medical diagnostic documents generated daily. These documents contain crucial patient information in various layouts and formats, making it difficult for healthcare systems to extract and utilize the data effectively. Traditional manual data entry methods are time-consuming, error-prone, and costly, while existing automated solutions often struggle to handle the diversity of document structures. To address this challenge, we propose a novel approach that focuses on extracting key information from medical diagnostic documents using a combination of Optical Character Recognition (OCR) and a Key Information Extraction (KIE) model. Note that in this study, we address the printed documents rather than handwritten documents. Our system is designed to automatically process and extract data from a wide range of document layouts and formats, enabling healthcare organizations to efficiently utilize the valuable information contained within these documents. While our model is capable of extracting various types of information from medical diagnosis documents, this research specifically focuses on six essential categories: doctor’s name, department name, hospital address, hospital name, diagnosis, and doctor’s comments.

By targeting these categories, our goal is to provide a comprehensive solution that captures the most critical and frequently required information for healthcare purposes.

**Figure 1:**
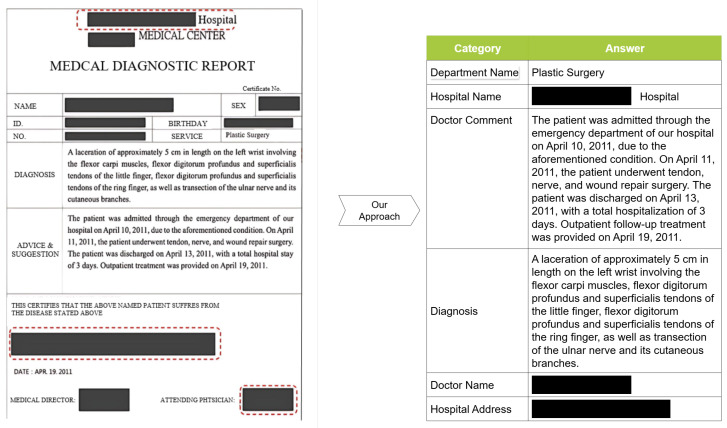
An illustrated example of a medical diagnosis document as well as the extracted information. (This is a synthetically generated document used for illustration purposes only. Certain fields containing identifiable information have been obscured. This represents the English translation. The original version employed in our study is in Chinese, as presented in Figure A1 of the Appendix A).

As illustrated in Figure 1, our approach comprises two main components: OCR and KIE models. The OCR module converts text from medical diagnosis documents into a machine-readable format, while the KIE module leverages advanced natural language processing techniques to identify and extract relevant information across six targeted categories. This integrated approach enables effective handling of the diverse layouts and formats of medical documents.

In response to the strict requirements for personal privacy in medical data, our research proposes a methodical approach to generate training data, ensuring compliance with privacy regulations while maintaining the integrity and utility of our dataset for medical document analysis. This process involves synthesizing realistic yet fictional doctor–patient information, including names, addresses, and medical details, using generative techniques. Effectively, we create a rich dataset that mirrors the diversity and complexity of real-world medical records without compromising individual privacy.

To evaluate the performance of our model, we conducted extensive experiments using a dataset of real medical diagnostic documents collected from the Internet. This dataset encompasses a wide range of document layouts and formats, providing a realistic representation of the challenges faced in processing unstructured medical data. We measured the accuracy and efficiency of our system in extracting information from the six categories using standard evaluation metrics such as precision, recall, and F1-score. By testing our model on real-world data, we assessed its effectiveness in handling the complexity and diversity of medical documents encountered in practice.

Furthermore, our research contributes to the field of medical informatics by addressing the need for effective solutions to handle the growing volume and complexity of unstructured medical data. The targeted approach of extracting information from six essential categories provides a focused and practical solution that can be readily adopted by healthcare organizations.

In the following sections, we will provide a detailed description of our methodology, including the OCR and document-understanding components, as well as the experimental setup and evaluation metrics used to assess the performance of our model. Finally, we will explore the implications of our research for the healthcare industry and outline potential future directions for enhancing and expanding our solution.

Existing methods, such as MedOCR [1], utilize Tesseract OCR [2] for text recognition, and employ BERT for natural language processing tasks. However, Tesseract OCR does not perform well on vertical text, and BERT considers only text embeddings, without accounting for layout or spatial information. To address these limitations, our method integrates text embeddings with spatial distance information, enabling the model to better understand the relationships between textual elements and their positions, thereby improving its information extraction performance. LayoutXLM is a multimodal pre-trained model designed for multilingual, visually rich document understanding. The model leverages the advantage of jointly learning text, layout, and image information end-to-end within a single framework. In this study, we employed vi-LayoutXLM, which utilizes only position embeddings and text embeddings, allowing the model to focus on learning the relationships between textual elements and their spatial distances within documents.

## 2. Related Work

In the field of OCR and NLP, combining different technologies has become an important trend. Tesseract OCR-RoBERTa, introduced by Zacharias et al. [2], combines Tesseract’s OCR abilities with RoBERTa’s language understanding skills. While it works well, the system mostly uses embeddings that only consider the text, which might miss the complex spatial arrangements often found in different document layouts.

In a similar way, MedOCR, developed by Zheng et al. [1], combines LayoutXLM with BERT to process document text. However, it faces difficulties when dealing with varied and complex document structures, which is a common problem when trying to work with many different layout types. These examples highlight the challenges faced by existing systems in adapting to the diversity and complexity of document layouts, particularly in the medical domain. Our research aims to address these limitations by proposing a novel approach that combines advanced OCR techniques with a document-understanding model to effectively extract key information from medical diagnostic documents, regardless of their layout or format.

In [3], the authors provide a comprehensive overview of deep learning approaches for table detection and structure recognition in document images, ranging from heuristic and machine learning methods to advanced deep neural networks. The authors systematically detail datasets (e.g., ICDAR, Marmot, TableBank); evaluation metrics (e.g., mAP, IoU); and deep learning architectures such as Faster R-CNN, Mask R-CNN, HRNet, and transformer-based models. The authors compare the performance of various methods on benchmark datasets, highlighting strengths and limitations, and organize open-source resources for researchers. The document also discusses key challenges, such as handling diverse table layouts and the lack of annotated data, and identifies future research directions, including improved generalization and end-to-end solutions.

In a systematic literature review, the authors of [4] comprehensively examined AI-based techniques for information extraction from unstructured documents, such as invoices, forms, and receipts, by introducing the evolution from manual and rule-based OCR methods to advanced AI approaches such as NLP and deep learning. In addition, the authors identified key challenges: unstructured data’s lack of standardization, poor-quality public datasets, and the absence of robust data validation techniques. However, while AI-based methods show strong potential for understanding and classifying complex document layouts, most current solutions remain template-dependent or domain-specific, limiting their scalability and generalizability. The authors proposed a framework for building high-quality, validated datasets and stressed the need for collaboration to address real-world complexities in unstructured data processing.

A comprehensive review of state-of-the-art techniques for extracting and structuring information from electronic medical texts—focusing on named-entity recognition, relation extraction, and section detection to meet the critical need for these technologies due to the increasing volume and complexity of medical records—is provided in [5]. The survey systematically compares rule-based, classical, and advanced deep learning approaches, including BERT, BiLSTM, CRF, and graph-based models, by leveraging contextual embeddings and transfer learning to achieve higher accuracy. Key challenges identified include medical language complexity, lack of data, privacy concerns, and the lack of benchmark datasets, particularly for non-English languages.

In a comprehensive review, the authors of [6] explored information extraction from electronic medical documents, focusing on named-entity recognition, relation extraction, and section detection using natural language processing and machine learning techniques. Similar to [5], the authors provided a survey on rule-based, dictionary-based, and machine learning approaches, discussing key challenges such as ambiguity, boundary detection, name variation, and composed entities. The review also provides an experimental analysis of state-of-the-art methods, lists benchmark datasets and supplementary resources, and identifies unresolved issues such as limited annotated corpora and privacy concerns.

A data verification approach for clinical registries is proposed in [7]. The authors made use of machine learning-enhanced OCR on digitized paper-based case report forms and applied NLP to extract electronic medical record data, and then they cross-verified both with registry entries. Compared with manual methods, this system achieved higher accuracy, better recall, and reduced elapsed time. The proposed methods are also effective at identifying missing and incorrect data. The authors concluded that this approach is efficient, scalable, and better than manual verification for enhancing registry data quality.

In [8], the authors present an end-to-end system for extracting and structuring biomedical knowledge from clinical notes by using BERT-based models coupled with CRF for named-entity recognition and relation extraction. The extracted knowledge graph enables intuitive analysis of drug interactions, side effects, and treatment rationales, with graph-based queries for clinical decision support.

In [9], the authors present a deep learning-based NLP data pipeline to accurately extract key clinical indicators, specifically the Apnea–Hypopnea Index and oxygen saturation, from scanned electronic health records of sleep study reports. The authors also systematically evaluate the effects of image preprocessing (grayscale, dilation/erosion, contrast adjustment); OCR with Tesseract; and multiple NLP models, including traditional machine learning and advanced deep learning (ClinicalBERT). ClinicalBERT combined with optimized image preprocessing achieved better performance. The study highlights the importance of incorporating document layout and structured inputs to improve extraction from scanned medical documents and offers a scalable solution to handle persistent challenges of scanned electronic health records.

An adaptive system for extracting and structuring information from medical report images by combining OCR and NLP techniques is presented in [10]. The authors introduce a genetic algorithm to optimize OCR hyperparameters, maximizing text extraction from diverse and low-quality documents. Considering the named-entity recognition task, extracted text is parsed with NLP to identify key entities such as tax IDs, payment dates, and costs. This approach demonstrated improved accuracy over previously published methods: Tesseract OCR and MedOCR.

## 3. Our Approach

### 3.1. Systems Architecture

**Figure 2:**
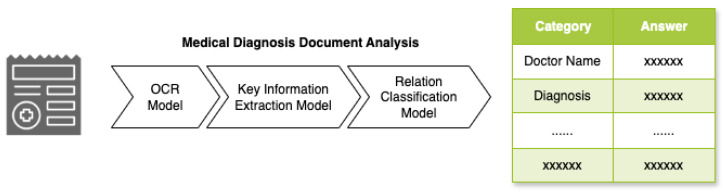
Overall architecture of the document-processing system.

We introduce a robust architecture designed to streamline the processing of complex documents, as illustrated in Figure 2. Our system comprises multiple interconnected models, each specialized for a specific aspect of document analysis. At the initial stage (Figure 2), the system employs an OCR model to detect and recognize text while determining precise text locations within the document. Building upon the OCR output, the Key Information Extraction (KIE) model identifies and extracts key–value pairs from the recognized text. In the final stage, the Relation Classification model analyzes the extracted key–value pairs to determine and classify relationships between different information elements within the document. This multi-model approach enables comprehensive document analysis, from text recognition to semantic understanding. Figure 2 provides a clear visualization of the information flow through these stages.

### 3.2. Data Collection and Generation

To address the privacy concerns inherent in medical data processing, we have developed a comprehensive approach for generating training data that represent real-world medical documents while preserving patient confidentiality.

**Figure 3:**
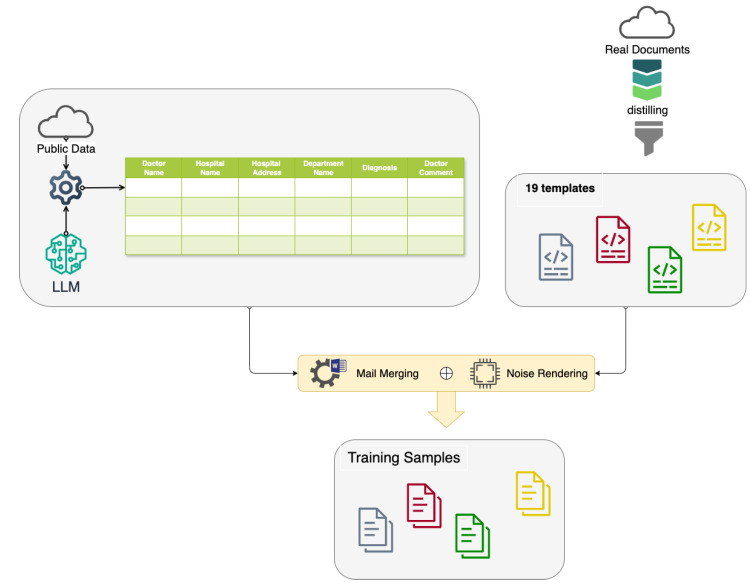
An illustration of generating training data.

As shown in Figure 3, we integrate the public data acquired from the Internet and the generated data from the LLM to prepare the key information to render training samples of medical diagnosis documents. In this study, the medical diagnosis document shown in Figure 1 identifies six crucial categories for the claim of health endurance: doctor name, doctor comment, department name, hospital name, hospital address, and diagnosis. The six-category data are organized as a structural file in the excel or csv format.

Meanwhile, we create **templates** from the real diagnosis documents. Given the word templates and source in the csv format associated with the supporting data, we make use of the **mail merge** function to generate training samples.

The template layout is deliberately not randomized; template designs are systematically structured to ensure both the realistic appearance and medical accuracy of the generated training data.

#### 3.2.1. Personal Information

We developed a synthetic data generation tool that creates privacy-preserving personal information: names are randomly generated from 1863 common traditional Chinese characters, ages from integers, gender from binary options (male/female), and identification numbers using a pattern of one alphabet plus five digits. This approach ensures dataset diversity while maintaining privacy.

#### 3.2.2. Hospital Information Acquisition

To enhance the authenticity of our dataset, we crawled real hospital data from the Taiwan Ministry of Health and Welfare’s website [11]. These data include hospital names, hospital addresses, and department names.

#### 3.2.3. Diagnosis-Related Data

We chose the top 100 most frequently reported ICD-10 codes from official health ministry sources [11] to cover a comprehensive range of medical conditions and diagnoses. This selection ensured that our dataset would represent a broad spectrum of healthcare scenarios in Taiwan.

#### 3.2.4. AI-Generated Doctor Comments

Utilizing the GPT-4 model by the OpenAI API [12], we implemented a prompting technique to generate context-appropriate doctor comments. This process produced medical insights tailored to each diagnosis, further enhancing the dataset’s realism and complexity.

**Figure 4:**
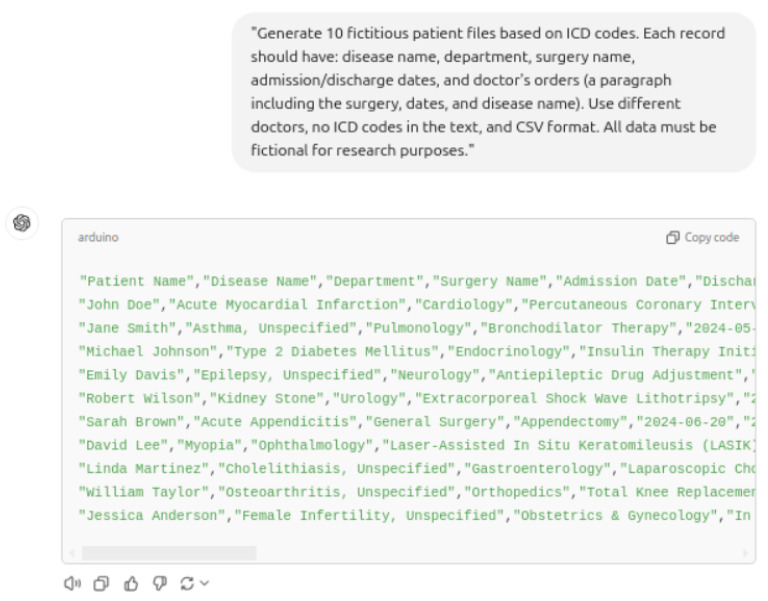
An example of the prompt and LLM-generated doctor’s comment. (This represents the English translation. The original version employed in our study is in Chinese, as presented in Figure A2 of the Appendix A.)

**Figure A2:**
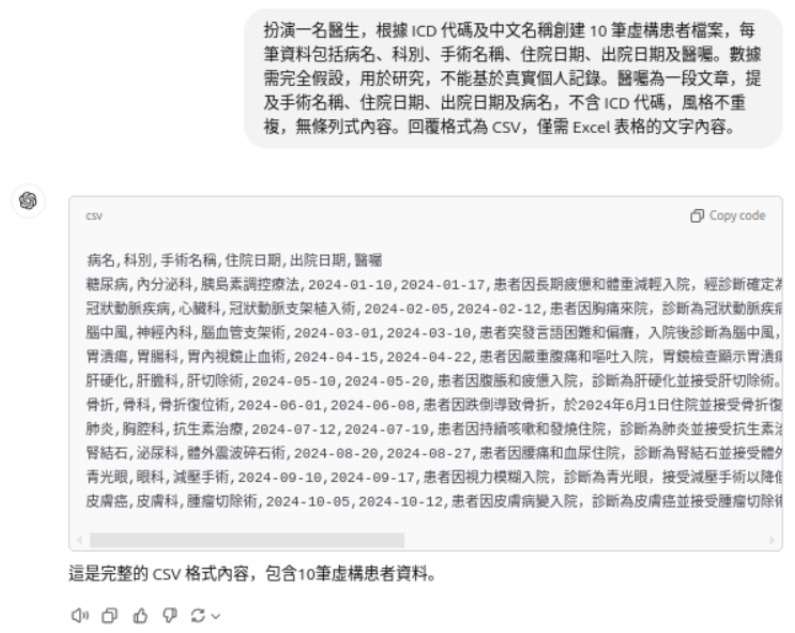
An example of the prompt and LLM-generated doctor’s comment (this represents the original version employed in our study in Chinese).

Figure 4 shows an example of the GPT-4 prompt as well as the generated doctor’s diagnosis comments for a given ICD-10 code. The research prompt is presented in Chinese. Figure 4 shows the English translation; Figure A2 shows the Chinese version.

#### 3.2.5. Document Image Rendering

**Figure 5:**
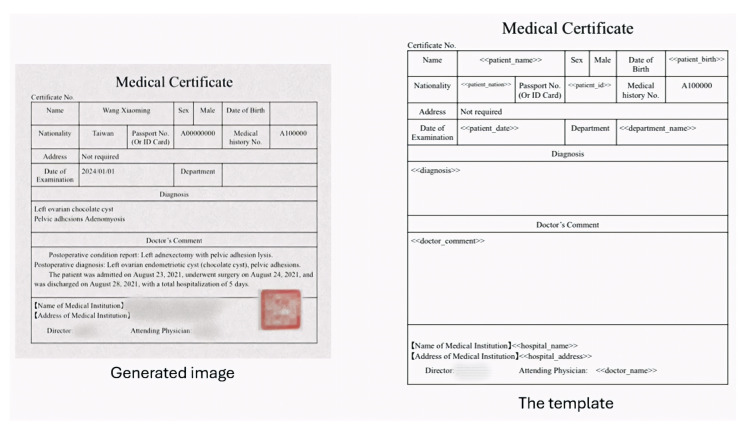
(**Left**): Generated image using our technique (this is a synthetically generated document used for illustration purposes only). (**Right**): The template (this represents the English translation; the original version employed in our study is in Chinese, as presented in Figure A3 of the Appendix A).

To simulate authentic hospital documents, several key steps were implemented in sequence. First, the document templates were created through establishing the **Main Document** in Word. Subsequently, bounding boxes were specified for each template using $\left(x_{1},y_{1}\right)$ and $\left(x_{2},y_{2}\right)$ coordinates, where each box contained a **Merge Field** corresponding to a specific category from the data source. The process continued with executing the **mail merge** function to produce training samples in bitmap image format. To achieve realistic document variation, Gaussian and uniform noise were applied to the rendered images, simulating the natural irregularities found in real-world documents. Figure 5 shows an example of a generated sample.

Data augmentation is generally advantageous for expanding the coverage and diversity of the training dataset, thereby enhancing the model’s overall robustness and performance. In our approach, we employed the data augmentation techniques using OpenCV, including Gaussian noise and salt-and-pepper noise. For text recognition, we also applied a variety of text-level augmentations: random character appearance, line and text structure variations, and visual effects or color adjustments. These augmentations increase the text recognition model’s ability to handle variations in character appearance, text layout, and visual noise, accordingly improving its extraction accuracy.

The final step involved formatting all annotations in accordance with the XFUND dataset specifications [13].

This approach ensures that our synthetic documents closely resemble real-world hospital records while preparing the dataset for document-understanding evaluations.

Our comprehensive data generation process, as illustrated in Figure 3, enables the creation of a privacy-compliant, diverse, and realistic dataset. By synthesizing various elements of medical documentation, we have developed a robust foundation for training and evaluating our OCR and information extraction system, while strictly adhering to ethical guidelines and privacy regulations in medical research.

Additionally, the problem addressed in this paper originates from a real-world application in the insurance industry. Currently, in this paper, we focus on Chinese documents. We collected as many authentic documents as possible to support our analysis. Based on these real data, we developed a set of templates for generating the training data. Since medical diagnosis documents cannot be arbitrarily designed, our templates are designed to encompass as many variations as possible. During the training data generation process, the list of hospital names is obtained from the publicly available datasets provided by the Ministry of Health and Welfare to closely simulate real-world cases. The “diagnosis” field is derived from the International Classification of Diseases (ICD) standard. These strategies collectively ensure both the realism and medical accuracy of the generated training data.

### 3.3. OCR Model

Our OCR model follows the architecture from PP-OCR [14], consisting of three main components: text detection, text box rectification, and text recognition. The PP-OCR model initially employed in this study was developed for Simplified Chinese. Therefore, the OCR model was retrained from scratch using a self-generated dataset in Traditional Chinese, and the “dictionary layer” was redefined to accommodate the Traditional Chinese character set.

**Figure 6:**
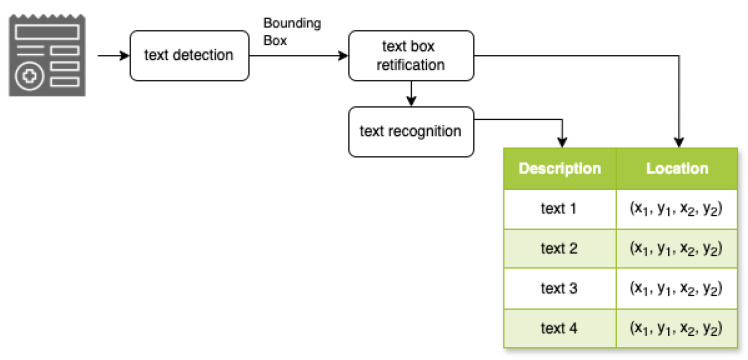
OCR model architecture.

As shown in Figure 6, the system operates in three sequential stages: *text detection model*, *text box rectification model*, and *text recognition model*. The text detection model transitions into text box rectification, where the system performs geometric transformations to normalize text orientations. This critical alignment process optimizes the subsequent recognition phase by ensuring uniform text positioning and orientation. In the final phase, the text recognition model processes the rectified regions, converting visual text elements into a machine-readable format through character-level analysis. This integrated approach ensures robust text extraction across diverse document types.

This structured approach allows for efficient and accurate extraction of textual information from various document layouts. By separating the process into these distinct stages, our model can handle diverse document types and text orientations, improving overall the OCR performance.

#### 3.3.1. Text Detection Model

For text detection, we adopt DBNet [15], an end-to-end segmentation-based algorithm proposed by Liao et al. We utilize a pre-trained model, which was initially trained on the ICDAR 2015 benchmark [16]. DBNet employs differentiable binarization for text detection, replacing traditional non-differentiable fixed binarization with an approximate binarization function that can be integrated into network training.

#### 3.3.2. Text Recognition Model

For text recognition, we employ two model architectures: Convolutional Recurrent Neural Networks (CRNNs) [17] and Patch-wise Image Tokenization Framework (SVTR) [18]. The CRNN combines Convolutional Neural Networks (CNNs) and Recurrent Neural Networks (RNNs). The CNN extracts features from the input image, while the RNN models sequential dependencies between characters. This combination allows the CRNN to effectively recognize text in various contexts, including different font sizes, styles, and orientations. The SVTR is an advanced model that uses both deep learning and traditional image processing techniques. It can recognize scene text in photographs with varying lighting, orientation, and skew. The SVTR also includes an attention mechanism, allowing the network to focus on the most important visual elements, thus improving its resistance to noise and distortion.

### 3.4. Extracting Key Information Model

Our research builds upon the VI-LayoutXLM model from PP-StructureV2 [19]. This model integrates layout-aware attention mechanisms with multiple embedding types: text, position, and visual embeddings. While sharing architectural similarities with LayoutXLM [20], the VI-LayoutXLM model distinguishes itself by incorporating visual embeddings, which are absent in the original LayoutXLM model. This multimodal approach enables effective fusion of textual and visual information for comprehensive document understanding.

A significant constraint in our study stems from the limited availability of document templates in existing datasets. This limitation makes purely visual-based methods less reliable for medical document processing. Therefore, we adopt the VI-LayoutXLM model with a visual-disabled approach for structured data extraction, which achieves high accuracy while requiring minimal training data.

### 3.5. Deep Mutual Learning

We adapt mutual learning [21] by implementing it across text detection, text recognition, and KIE components. For each component, we employ two parallel neural networks ($\Theta_{1}$ and $\Theta_{2}$), with the following total loss functions:

(1) $$L_{\Theta 1_{i}}=L_{C1_{i}}+D_{KL}\left(p_{2_{i}}\;\|\;p_{1_{i}}\right)$$

(2) $$L_{\Theta 2_{i}}=L_{C2_{i}}+D_{KL}\left(p_{1_{i}}\;\|\;p_{2_{i}}\right)$$

where $L_{C1}$ and $L_{C2}$ are each model’s loss, and $D_{KL}$ represents the KL divergence between the networks’ predictions, in the *i*-th epoch.

### 3.6. Relation Classification Model

**Figure 7:**
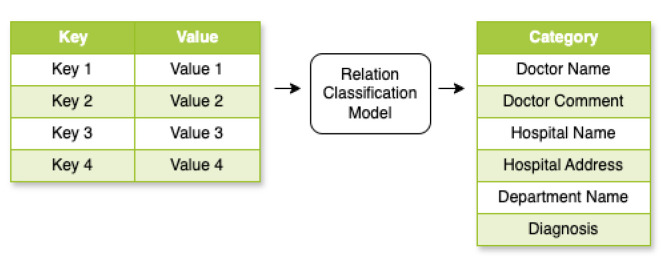
Relation Classification model framework.

As illustrated in Figure 7, the Relation Classification model maps key–value pairs $\left(k_{i},v_{i}\right)$ from the KIE output to a predefined relation set $R=r_{1},\ldots ,r_{m}$. In our application, *R* is the set of six categories. Through transformer embeddings in $R^{d}$ space, pairs undergo transformation via embedding functions and transformer layers. We adapted this idea from Tian et al.’s work [22] from the model output probability distributions over *R*, determining the most likely relation type for each pair.

In developing our relation extraction (RE) models, we built upon the advanced transformer architectures provided by the HuggingFace Transformers library [23] and PyTorch [24]. Initially, we engaged with transformer models pre-trained on general English text available in the HuggingFace repository as a reference model. To refine our approach for the medical domain, we selected the specialized models Bio-BERT [25] and Clinical-Longformer [26], both renowned for their pre-training on the English MIMIC-III dataset [27], a rich source of clinical narratives.

Despite the linguistic differences between our Traditional Chinese dataset and the English MIMIC-III corpus, both datasets are unified by their intrinsic knowledge of the clinical domain. This shared medical context underpins our decision to fine-tune Bio-BERT and Clinical-Longformer with our dataset. We began with the pre-trained BERT-base-Chinese tokenizer and model. To better support Chinese biomedical text, we extended the vocabulary by adding domain-specific Chinese biomedical terms so that these specialized terms would be treated as meaningful tokens rather than simply broken into sub-words or unknown tokens. For each newly added token, we randomly initialized the corresponding embedding vector while retaining the original pre-trained weights for existing tokens. We then fine-tuned the adapted model on our annotated Chinese biomedical dataset, allowing the model to learn and leverage the new tokenization and vocabulary for improved performance in the biomedical domain. The fine-tuning process was tailored to bridge the language gap while capitalizing on the domain-specific insights that these models have acquired through their initial pre-training. This involved adapting the models to better capture the nuances of clinical information presented in Traditional Chinese, leveraging their pre-existing understanding of clinical contexts derived from MIMIC-III.

Our fine-tuning regimen was structured around a comprehensive five-fold cross-validation strategy, aimed at optimizing hyperparameters such as training epochs and batch sizes, against the backdrop of a consistent learning rate of $1\times 10^{-5}$ and a fixed random seed of 13. The selection of the optimal model configuration was guided by the highest micro-averaged strict F1-scores obtained during cross-validation.

Integrating and fine-tuning Bio-BERT and Clinical-Longformer for our Traditional Chinese dataset represents a strategic pivot towards leveraging sophisticated AI technologies to improve Relation Classification in clinical texts across languages. This methodology not only benefits from the domain-specific pre-training of these advanced transformer models on the MIMIC-III corpus but also innovatively adapts them to manage the complexities and specificities of medical narratives in a different language, thereby enhancing their utility and accuracy in extracting meaningful relationships from clinical documents.

## 4. Evaluation

### 4.1. Evaluation Metrics

We employ specific metrics for each task in our evaluation process:

For text detection, we apply the *HmeanIOU* Metric, which is the harmonic mean of Intersection over Union (IOU) in terms of precision (*P*) and recall (*R*). This metric effectively balances detection accuracy and completeness.

The formula is given as follows:

(3) $$HmeanIOU=\frac{2PR}{P+R}$$

where precision (*P*) represents the proportion of correctly detected text regions, and recall (*R*) reflects the proportion of ground-truth text regions successfully detected.

For text recognition, we apply the *Character Error Rate* (CER) metric, which measures the accuracy of recognized text at the character level. The Character Error Rate is defined as follows:

(4) $$CER=\frac{S+D+I}{N}$$

where *S* is the number of substituted characters, *D* is the number of deleted characters, *I* is the number of inserted characters, and *N* is the total number of characters in the ground truth.

For Relation Classification, we use the word-level F1-score. These metrics are calculated as follows:

(5) $$\mathrm{Precision}=\frac{\mathrm{Correct}\;\mathrm{matches}}{\mathrm{Number}\;\mathrm{of}\;\mathrm{detected}\;\mathrm{words}}$$

(6) $$\mathrm{Recall}=\frac{\mathrm{Correct}\;\mathrm{matches}}{\mathrm{Number}\;\mathrm{of}\;\mathrm{ground}\;\mathrm{truth}\;\mathrm{words}}$$

(7) $$F1-Score=2\times \frac{\mathrm{Precision}\times \mathrm{Recall}}{\mathrm{Precision}+\mathrm{Recall}}$$

For Key Information Extraction, we use two sub-metrics: (1) **Semantic Entity Labeling**, where text segments are categorized as keys, values, or other entities, and (2) **Entity Linking**, where relationships between keys and values are predicted using a binary classification model.

Both tasks are evaluated using precision, recall, and F1-Score, as follows:

**Semantic Entity Labeling:** Each entity is assigned a category, and performance is measured by

(8) $$\mathrm{Precision}=\frac{TP}{TP+FP}$$

(9) $$\mathrm{Recall}=\frac{TP}{TP+FN}$$

(10) $$F1-Score=2\times \frac{\mathrm{Precision}\times \mathrm{Recall}}{\mathrm{Precision}+\mathrm{Recall}}$$

where TP (True Positives) are correctly labeled key/value entities, FP (False Positives) are incorrectly labeled entities, and FN (False Negatives) are missed key/value entities.

**Entity Linking:** To evaluate the correctness of key–value relationships, we define

(11) $$\mathrm{Precision}=\frac{\mathrm{Correct}\;\mathrm{Links}\;\mathrm{Predicted}}{\mathrm{Total}\;\mathrm{Links}\;\mathrm{Predicted}}$$

(12) $$\mathrm{Recall}=\frac{\mathrm{Correct}\;\mathrm{Links}\;\mathrm{Predicted}}{\mathrm{Total}\;\mathrm{Ground}\;\mathrm{Truth}\;\mathrm{Links}}$$

(13) $$F1\text{-}\mathrm{Score}=2\times \frac{\mathrm{Precision}\times \mathrm{Recall}}{\mathrm{Precision}+\mathrm{Recall}}$$

These metrics ensure an accurate evaluation of both entity classification and their corresponding relationships, providing a structured framework for key–value pair extraction.

For Relation Classification, we also use precision, recall, and F1-score. These metrics are calculated for each type of relation.

### 4.2. Dataset

We generated 5600 training data samples based on the first 100 ICD-10 codes and collected 66 images from the Internet for testing, as shown in Table 1. All datasets are in Traditional Chinese and have been annotated with respect to the six categories: doctor name, hospital name, hospital address, diagnosis, doctor comment, and department name.

It is a significant challenge to collect the medical diagnosis documents (certificate of diagnosis, real data). To the best of our ability, we obtained real data from publicly available sources on the Internet. To the best of our knowledge, there is no public benchmark of Chinese medical diagnosis documents.

## 5. Results

For the efficiency evaluation, we trained the text detection and text recognition model for 500 epochs. For the LayoutXLM and BERT models, we trained for 100 epochs each. The elapsed time for training the models was approximately 72 h. The elapsed time for generating the training data was approximately 2 h. The average elapsed time for testing (inference) was 15 s per case. The experiments were run using an NVIDIA RTX A6000 workstation GPU, featuring 48 GB GDDR6 memory, 10,752 CUDA cores, and a memory bandwidth of up to 768 GB/s.

With respect to the effectiveness study, our OCR model was evaluated in two main areas: text detection and text recognition. The performance was assessed using standard metrics (loss and accuracy) for both tasks. The following tables summarize the results.

### 5.1. Text Detection

Our text detection experiments utilized DBNet as the base architecture, which was pre-trained by Baidu on the ICDAR 2015 dataset. We evaluated the performance using two different backbone networks—MobileNetV3 and ResNet50—with an initial learning rate of 0.01 and a batch size of 32, for 500 epochs on our dataset. The model was optimized using the Adam and HmeanIOU metrics.

In Table 2, the experimental results demonstrate that MobileNetV3 achieves superior performance compared with ResNet50, with higher scores on both the training and evaluation sets. MobileNetV3 reaches 0.99 and 0.97 on the training and evaluation sets, respectively, while ResNet50 achieves 0.95 and 0.94, respectively.

### 5.2. Text Recognition

This section evaluates two text recognition models: SVTR (Spatial-Visual Text Representation) and CRNN (Convolutional Recurrent Neural Network).

The experimental results in Table 3 show distinct performance patterns for both models. In training performance, SVTR achieved a higher accuracy with 0.97, while CRNN showed a slightly lower accuracy with 0.95. For the evaluation performance, CRNN demonstrated better generalization with 0.93 accuracy, while SVTR showed performance degradation to 0.89.

Analysis shows that while SVTR excels in fitting training data, CRNN provides better generalization to unseen data. The 0.08 performance drop in SVTR (0.97 to 0.89), versus CRNN’s 0.02 drop (0.95 to 0.93), suggests potential overfitting in SVTR. CRNN’s more stable performance across both datasets makes it more suitable for practical applications, where consistent performance on diverse, unseen text is crucial.

These findings suggest that CRNN is the more reliable choice for real-world text recognition tasks, particularly when generalization capability is prioritized over training set performance.

### 5.3. Key Information Extraction Model

Table 4 presents a simplified comparative performance analysis of two Key Information Extraction models: LayoutXLM and VI-LayoutXLM. These models were evaluated in two critical tasks, Semantic Entity Recognition (SER) and Relation Classification (RC), using both training and validation datasets.

In the SER task, VI-LayoutXLM demonstrated superior performance on the training set, with an accuracy of 0.92, compared with LayoutXLM’s 0.85. However, LayoutXLM showed better generalization to the validation set, achieving 0.9 accuracy compared with VI-LayoutXLM’s 0.88. This suggests that VI-LayoutXLM may be overfitting to the training data for the SER task. For the RE task, VI-LayoutXLM outperformed LayoutXLM in both the training and validation sets. VI-LayoutXLM achieved accuracies of 0.9 and 0.88 on the training and validation sets, respectively, while LayoutXLM scored 0.88 and 0.85, respectively. This indicates that VI-LayoutXLM has better overall performance and generalization capabilities for relation extraction. The results suggest that VI-LayoutXLM might be more suitable for applications prioritizing relation extraction, while LayoutXLM shows promise in Semantic Entity Recognition, particularly on unseen data. Further investigation into the models’ architectures and training procedures could provide insights into improving VI-LayoutXLM’s generalization for SER tasks and enhancing LayoutXLM’s performance in RE tasks.

### 5.4. Relation Classification Model

Table 5 presents the performance metrics for two Relation Classification models: Bio-BERT and Clinical-Longformer [25]. These models were evaluated for their ability to extract relational data from clinical narratives, using precision, recall, and the F1-score as key performance indicators.

We used the micro-averaged precision, recall, and F1-score aggregated from all relation categories, following the standard evaluation approach for clinical Relation Classification (RE).

The Clinical-Longformer also exhibited strong performance, with consistently high scores across all metrics. It achieved a precision of 0.949, recall of 0.948, and an F1-score of 0.948. These balanced scores suggest that the Clinical-Longformer maintains a good equilibrium between correctly identifying relevant relationships (precision) and capturing a high proportion of all relevant relationships in the text (recall). Comparing the two models, we can observe that the Bio-BERT slightly outperforms the Clinical-Longformer, particularly regarding recall and F1-score. However, Bio-BERT shows marginally higher precision. This discrepancy in the performance metrics probably reflects the inherent differences in the model architectures and the training approaches. Bio-BERT’s exceptionally high recall (0.98) suggests that it is particularly effective at identifying a wide range of relevant relationships, potentially capturing more complex or nuanced associations in the clinical narratives. Its slightly lower precision (0.90) compared with its recall indicates that it may occasionally overpredict relationships, but this is offset by its comprehensive coverage. Clinical-Longformer’s balanced precision and recall scores (both 0.949) indicate a very consistent performance in both accurately identifying relationships and capturing a high proportion of relevant relationships. These results underscore the effectiveness of both models in accurately extracting relational data from clinical narratives. The choice between these models may depend on specific application requirements, with Bio-BERT potentially being preferred for tasks requiring high recall, and Clinical-Longformer for applications where balanced precision and recall are crucial.

### 5.5. End-to-End System Evaluation

Our approach integrates the best-performing components of each stage of the document-processing pipeline. In text detection, we use the MobileNetV3 backbone, which showed lower loss on the evaluation set. For text recognition, we employ the CRNN model, demonstrating better generalization, with 0.93 accuracy on the evaluation set. Key Information Extraction is handled by VI-LayoutXLM, which excelled in Relation Classification tasks. Finally, Relation Classification is performed by Bio-Longformer, which achieved the highest F1-score of 0.98. This combination of top-performing elements results in a robust system that excels across various document-processing tasks. The integration of these specialized components enables comprehensive processing of medical documents, from initial text detection through to final information extraction and classification.

The results from Table 6 indicate that our approach consistently outperforms both TesseractOCR-Roberta [2] and Medocr [1] in most categories. Notably, our model achieves the highest accuracy in extracting doctor names (0.88), diagnoses (0.99), doctor comments (0.95), hospital names (0.90), and hospital addresses (0.93). The performance in the department name category is tied with that of Medocr, with both achieving an accuracy of 0.90.

A review of the error cases within the test set reveals several recurrent issues that lead to incorrect detection. First, the text is not fully arranged, in a form that prevents the KIE system from accurately identifying complete paragraphs. Second, portions of the text are obscured by overlapping stamps. Third, some test images exhibit insufficient resolution.

### 5.6. Ablation Study with Deep Mutual Learning

We conducted ablation experiments incorporating DML across our three main components. The aim was to examine performance with (+) and without (–) DML integration, as shown in Table 7.

## 6. Conclusions

This study faces several limitations that could affect its scope and applicability. Notably, the absence of open-source clinical datasets in Traditional Chinese limited our ability to comprehensively represent the spectrum of medical conditions. Furthermore, the sensitive nature of human and hospital-related information posed significant challenges in data collection, affecting the depth of personal and institutional data included in our dataset. These limitations underscore the need for cautious interpretation of our findings and suggest directions for future research to overcome these challenges.

Our findings reveal that transformer models, when applied to the relational extraction task within Key Information Extraction (KIE) systems, achieve higher accuracy compared with traditional approaches. This success can be attributed to the transformer’s inherent ability to analyze and predict relationships within key–value pairs, focusing precisely on the structured data typical of KIE models. However, the challenges of accessing comprehensive real-world datasets and the model’s performance on unstructured information underscore the need for ongoing refinement and adaptation of these models.

The problem addressed in this paper originates from a real-world application in the insurance industry. Ideally, given sufficient real-world data, the dataset would be divided into training and testing subsets. However, obtaining real data that contain confidential or sensitive personal information is highly challenging. Therefore, we developed a training data generation process to address this issue. This process employs a set of templates, integrated with authentic hospital lists and the International Classification of Diseases (ICD) standard, to ensure both realism and medical accuracy. Furthermore, every genuine document includes a hospital name that appears in the official list published by the Ministry of Health and Welfare. From this perspective, the case might not be regarded as an instance of data leakage. Moreover, we can rigorously exclude hospital names present in the testing set from the candidate list used during the generation procedure. Nevertheless, from another perspective, the possibility of data leakage cannot be entirely excluded.

The proposed method is subject to several limitations, including the restricted size of the available dataset, variability in certificate formats and layouts, multi-language challenges, risks of data leakage, difficulties with handwritten characters, and the presence of stamps or stains. Addressing these limitations represents a primary direction for future research, aiming to expand the transformer’s utility and efficacy in extracting and classifying relationships across a wider array of data formats and contexts, thereby enhancing the robustness and applicability of KIE systems in diverse medical informatics applications.

## Figures and Tables

**Table 1:** Data distribution.

Category	Training Data	Testing Data
Number of images	5600	66
Templates	19	N/A
OCR annotation	29,470	255

**Table 2:** Text detection performance metrics.

Backbone	Training Set	Testing Set
MobibleNetV3	0.99	0.97
RestNet50	0.95	0.94

**Table 3:** Text recognition performance metrics.

Model	Training Set	Testing Set
SVTR	0.97	0.89
CRNN	0.95	0.93

**Table 4:** Comparative performance of Vi-LayoutXLM and LayoutXLM.

Model	SER		RE	
	Training	Validation	Training	Validation
LayoutXLM	0.85	0.90	0.88	0.85
Vi-LayoutXLM	0.92	0.88	0.90	0.88

**Table 5:** Evaluation of Relation Classification models.

Model	Precision	Recall	F1-Score
Bio-BERT	0.900	0.980	0.938
Clinical-Longformer	0.949	0.948	0.948

**Table 6:** Comparative performance on key categories.

Category	TOCR-Roberta	Medocr	Our Method
Doctor Name	0.22	0.80	0.88
Diagnose	0.45	0.95	0.99
Doctor Comment	0.50	0.82	0.95
Department Name	0.45	0.90	0.90
Hospital Name	0.72	0.80	0.90
Hospital Address	0.61	0.85	0.93

**Table 7:** Component performance comparison with and without DML.

Component	Model	Difference
Text Detection	DBNet+MobilenetV3	±20.6
	DBNET+Restnet50	±12.7
Text Recognition	CRNN	±3.0
	SVTR	±2.5
KIE	LayoutXLM	±5.0
	VI-LayoutXLM	±7.0

**Figure A1:**
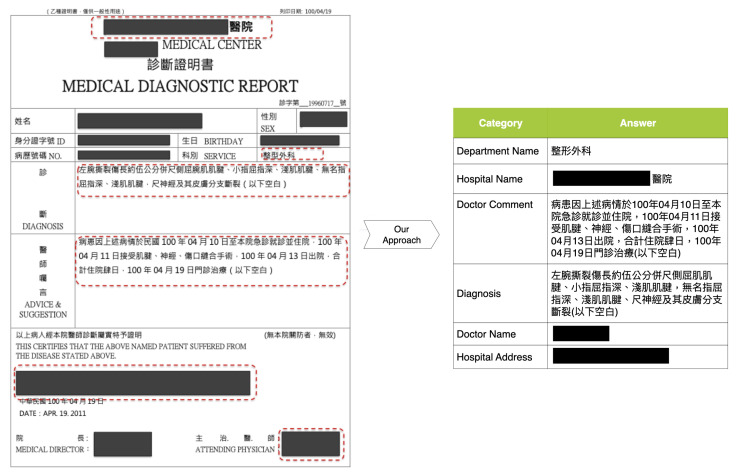
An illustrated example of a medical diagnosis document as well as the extracted information. (This is a synthetically generated document used for illustration purposes only. Certain fields containing identifiable information have been obscured.)

**Figure A3:**
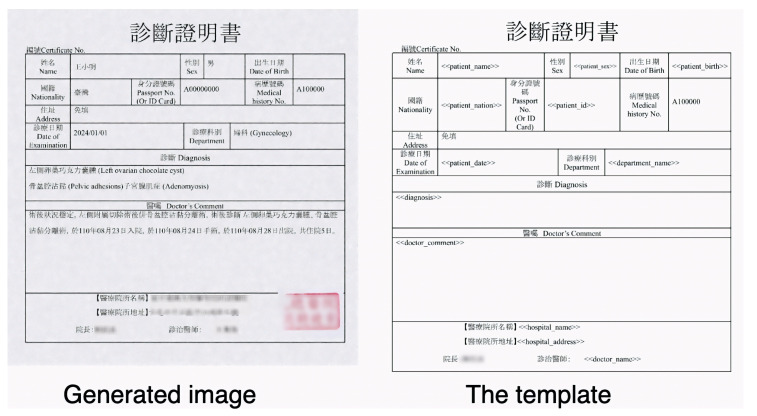
(**Left**): Generated image using our technique (this is a synthetically generated document used for illustration purposes only). (**Right**): The template.

## Data Availability

The original data presented in this study, including the template files, are openly available at https://reurl.cc/G5Rk2G. The testing dataset, consisting of 66 medical diagnostic documents collected from publicly available Internet sources, is available upon request for research purposes. The source code corresponding to our proposed methodology is accessible at https://github.com/danghoangnhan/XFUND_generator for the training data generation component, and at https://reurl.cc/nlEZ5X for the testing module.

## Appendix A

Figure A1–Figure A3 display the original Chinese versions of the figures referenced in our study.
